# Supplementary material for: Fixation dynamics on hypergraphs
Source: PLoS Comput Biol. 2023 Sep 26;19(9):e1011494. doi: 10.1371/journal.pcbi.1011494 (PMC10558078; doi:10.1371/journal.pcbi.1011494)
Supplement: S1 Text — Fig A. Fixation probability for the star 3-uniform hypergraph with N = 1500. We compare it with the fixation probability for the Moran process. The inset on the left magnifies the result for r values smaller than and close to r = 1. The inset on the right magnifies the result for r values greater than and close to r = 1. In the main plot, the result for the Moran process, shown by the black line, is not identical but close to that for the star 3-uniform hypergraph, shown by the green line, such that the former is hidden behind the latter. Fig B. Fixation probability for the weighted one-mode projection of star 3-uniform hypergraphs. We compare it with the fixation probability for the Moran process and star 3-uniform hypergraphs. (A) N = 4. (B) N = 5. (C) N = 20. (D) N = 200. The insets in (C) and (D) magnify the results for r values smaller than and close to r = 1. In the inset in (D), the results for the star 3-uniform hypergraph (shown by the blue line) and the Moran process (shown by the black line) are close to that for the one-mode projection (shown by the orange line) such that the blue and the black lines are almost hidden behind the orange line. In (A), (B), and the main panel of (D), the results for the Moran process (shown by the black lines) are not identical but close to those for the one-mode projection (shown by the orange lines) such that the former are hidden behind the latter. Fig C. Initial position of the two nodes of type A on the cyclic 3-uniform hypergraph. (A) The two nodes of type A do not share any hyperedge. (B) The two nodes of type A share two hyperedges. (C) The two nodes of type A share one hyperedge. Text A. Fixation probabilities for small 3-uniform hypergraphs. Text B. Proof of Eq (22) for N = 4. Text C. Derivation of the entries of the transition probability matrix for i ∈ {2, …, N − 1} for the cyclic 3-uniform hypergraph under model 1. Text D. Proof of Eq (27) for N = 4. Text E. Proof of Eq (31) for i = N − 3 and i = N − 2. Text F. [file pcbi.1011494.s001.pdf]

Supporting Information for: Fixation dynamics on hypergraphs

Ruodan Liu, Naoki Masuda

Text A. Fixation probabilities for small 3-uniform hypergraphs

A.1 Fixation probabilities for small 3-uniform hypergraphs under model 1

For the complete 3-uniform hypergraph with $N = 4$, we obtain

$$x_2 = \frac{r(3r^2+8r+1)}{3(r+1)^3}, \tag{S1}$$

$$x_3 = \frac{r(r+2)}{(r+1)^2}. \tag{S2}$$

For the complete 3-uniform hypergraph with $N = 5$, we obtain

$$x_2 = \frac{r^2(8r^2+26r+8)}{8r^4+28r^3+33r^2+28r+8}, \tag{S3}$$

$$x_3 = \frac{r(8r^3+28r^2+25r+2)}{8r^4+28r^3+33r^2+28r+8}, \tag{S4}$$

$$x_4 = \frac{4r(2r^3+7r^2+8r+4)}{8r^4+28r^3+33r^2+28r+8}. \tag{S5}$$

For the cyclic 3-uniform hypergraph with $N = 4$, we obtain

$$x_2 = \frac{r(3r^2+8r+1)}{3(r+1)^3}, \tag{S6}$$

$$x_3 = \frac{r(r+2)}{(r+1)^2}. \tag{S7}$$

For the cyclic 3-uniform hypergraph with $N = 5$, we obtain

$$x_2 = \frac{6r^2(r^2+3r+1)}{6r^4+20r^3+23r^2+20r+6}, \tag{S8}$$

$$x_3 = \frac{r(6r^3+20r^2+17r+2)}{6r^4+20r^3+23r^2+20r+6}, \tag{S9}$$

$$x_4 = \frac{2r(3r^3+10r^2+11r+6)}{6r^4+20r^3+23r^2+20r+6}. \tag{S10}$$

For the star 3-uniform hypergraph with $N = 4$, we obtain

$$x_2 = \frac{r(3r^2+7r+1)}{3r^3+8r^2+8r+3}, \tag{S11}$$

$$x_3 = \frac{3r(3r+5)}{4(3r^2+5r+3)} + \frac{9r(r^2+2r+1)}{4(9r^3+18r^2+14r+3)}. \tag{S12}$$

For the star 3-uniform hypergraph with $N = 5$, we obtain

$$x_2 = \frac{2r^2(144r^3+548r^2+512r+71)}{5(144r^5+620r^4+1040r^3+1055r^2+750r+216)} + \frac{3r^2(72r^2+196r+72)}{5(72r^4+202r^3+217r^2+202r+72)}, \tag{S13}$$

$$x_3 = \frac{3r(72r^3+202r^2+145r+6)}{5(72r^4+202r^3+217r^2+202r+72)} + \frac{12r(36r^4+125r^3+164r^2+88r+12)}{5(216r^5+750r^4+1055r^3+1040r^2+620r+144)}, \tag{S14}$$

$$x_4 = \frac{4r(72r^3+202r^2+213r+108)}{5(72r^4+202r^3+217r^2+202r+72)} + \frac{8r(36r^4+110r^3+133r^2+110r+36)}{5(288r^5+880r^4+1070r^3+1025r^2+490r+72)}. \tag{S15}$$

A.2 Fixation probabilities for small 3-uniform hypergraphs under model 2

We obtain $x_1 = 0$ and $x_{N-1} = 1$ for any 3-uniform hypergraph under model 2. Therefore, when $N = 4$, only x_2, which we show in the main text, is nontrivial. When $N = 5$, only x_2 and x_3 are nontrivial. Because we show x_2 in the case of $N = 5$ in the main text, here we show x_3 in the case of $N = 5$. For the cyclic 3-uniform hypergraph with $N = 5$, we obtain

$$x_3 = \frac{r^2-1}{2(r^2-r^{-1})} + \frac{1}{2(1+4r)}\left(4r + \frac{r-1}{r-r^{-2}}\right). \tag{S16}$$

For the star 3-uniform hypergraph with $N = 5$, we obtain

$$x_3 = \frac{3r(r+3)}{5(r^2+3r+1)} + \frac{2r(4r^2+15r+4)}{5(4r^3+15r^2+13r+3)}. \tag{S17}$$

Text B. Proof of Eq. (22) for $N = 4$

If $N = 4$, then $v_{\ell-2}$ and $v_{\ell+2}$ are identical. In this case, there are two sequences of events through which the state moves from $i = 1$ to $i = 0$ in one time step. In the first sequence, $v_{\ell-2}$, which is of type B, is selected as parent with probability $1/(r+3)$. Then, the hyperedge that contains the parent and v_ℓ, i.e., $\{\ell-2, \ell-1, \ell\}$ or $\{\ell, \ell+1, \ell-2\}$, is used for reproduction, which occurs with probability $2/3$. In the second sequence, either $v_{\ell-1}$ or $v_{\ell+1}$ is selected as parent, which occurs with probability $2/(r+3)$. Then, one of the two hyperedges that contain the parent and v_ℓ is used for reproduction, which occurs with probability $2/3$. For example, if $v_{\ell-1}$ is selected as parent and hyperedge $\{\ell-2, \ell-1, \ell\}$ or $\{\ell-1, \ell, \ell+1\}$ is used for reproduction, then the state moves from 1 to 0. By summing up these probabilities, we obtain Eq. (22).

Text C. Derivation of the entries of the transition probability matrix for $i \in \{2, \ldots, N-1\}$ for the cyclic 3-uniform hypergraph under model 1

In this section, we derive the (i, j) entries of the transition probability matrix for the cyclic 3-uniform hypergraph under model 1, where $i \in \{2, \ldots, N-1\}$.

Similarly to the case of $i = 1$, when $i = N-1$, there are three types of events that can occur in a time step. Without loss of generality, we assume that the ℓth node is the only node of type B (see Fig 3B for a schematic). The state moves from $i = N-1$ to $i = N-3$ whenever v_ℓ is selected as parent, which occurs with probability $1/[r(N-1)+1]$. Therefore, we obtain

$$p_{N-1,N-3} = \frac{1}{r(N-1)+1}, \tag{S18}$$

which is Eq. (26) in the main text. Alternatively, the state moves from $i = N-1$ to $i = N$ such that type A fixates in the following two cases. In the first case, either node $v_{\ell-2}$ or $v_{\ell+2}$, which is of type A, is selected as parent. If $N \geq 5$, these two nodes are distinct. Therefore, this event occurs with probability $2r/[r(N-1)+1]$. Then, the hyperedge that contains the parent and v_ℓ is used for reproduction, which occurs with probability $1/3$. In the second case, either $v_{\ell-1}$ or $v_{\ell+1}$ is selected as parent, which occurs with probability $2r/[r(N-1)+1]$. Then, one of the two hyperedges that contain the parent and v_ℓ is used for reproduction, which occurs with probability $2/3$. By summing up these probabilities, we obtain

$$p_{N-1,N} = \frac{2r}{r(N-1)+1}, \tag{S19}$$

which is Eq. (27) in the main text. In fact, Eq. (S19) also holds true for $N = 4$; see Text D for the proof. If any other event occurs, then $i = N - 1$ remains unchanged. Therefore, we obtain

$$p_{N-1,N-1} = 1 - p_{N-1,N-3} - p_{N-1,N} = \frac{r(N-3)}{r(N-1)+1}, \tag{S20}$$

$$p_{N-1,j} = 0 \text{ if } j \neq N-3, N-1, N, \tag{S21}$$

which are Eqs. (28) and (29), respectively, in the main text.

When $i \in \{2, \ldots, N-2\}$, there are five types of possible events. Without loss of generality, we assume that the ℓth to the $(\ell + i - 1)$th nodes are of type A and that all other nodes are of type B (see Fig 3C for a schematic). If $\ell + i - 1$ is larger than N, then we interpret $\ell + i - 1$ as the number modulo N (i.e., $\ell + i - 1 - N$); the same convention applies in the following text. In the first type of event, either node $v_{\ell-1}$ or $v_{\ell+i}$, which is of type B, is selected as parent; this event occurs with probability $2/(ri + N - i)$. Then, the hyperedge that contains the parent and two nodes of type A is used for reproduction, which occurs with probability 1/3. In this case, the state i decreases by two. For example, if $v_{\ell-1}$ is selected as parent and hyperedge $\{\ell-1, \ell, \ell+1\}$ is selected, then the state moves from i to $i-2$. Therefore, we obtain

$$p_{i,i-2} = \frac{2}{3(ri + N - i)}, \tag{S22}$$

which is Eq. (30) in the main text. In the second type of event, either $v_{\ell-2}$, $v_{\ell-1}$, $v_{\ell+i}$, or $v_{\ell+i+1}$, which is of type B, is selected as parent. If $i \leq N - 4$, these four nodes are distinct. Therefore, this event occurs with probability $4/(ri + N - i)$. Then, the hyperedge that contains the parent, a node of type B, and a node of type A is used for reproduction, which occurs with probability 1/3. In this case, the state i decreases by one. For example, if $v_{\ell-2}$ is selected as parent and hyperedge $\{\ell-2, \ell-1, \ell\}$ is used for reproduction, then the state moves from i to $i-1$ because v_ℓ turns from A to B. Therefore, we obtain

$$p_{i,i-1} = \frac{4}{3(ri + N - i)}, \tag{S23}$$

which is Eq. (31) in the main text. In fact, Eq. (S23) also holds true for $i = N - 3$ and $i = N - 2$ although some of $v_{\ell-2}$, $v_{\ell-1}$, $v_{\ell+i}$, and $v_{\ell+i+1}$ are identical nodes when $i = N - 3$ or $i = N - 2$; see Text E for the proof. In the third type of event, either v_ℓ, $v_{\ell+1}$, $v_{\ell+i-2}$, or $v_{\ell+i-1}$, which is of type A, is selected as parent. If $i \geq 4$, these four nodes are distinct. Therefore, this event occurs with probability $4r/(ri + N - i)$. Then, the hyperedge that contains the parent node, a node of type A, and a node of type B is used for reproduction, which occurs with probability 1/3. In this case, state i increases by one. For example, if v_ℓ is selected as parent and hyperedge $\{\ell-1, \ell, \ell+1\}$ is used for reproduction, then the state moves from i to $i+1$ because $v_{\ell-1}$ turns from B to A. Therefore, we obtain

$$p_{i,i+1} = \frac{4r}{3(ri + N - i)}, \tag{S24}$$

which is Eq. (32) in the main text. In fact, Eq. (S24) also holds true for $i = 2$ and $i = 3$ although some of v_ℓ, $v_{\ell+1}$, $v_{\ell+i-2}$, and $v_{\ell+i-1}$ are identical nodes when $i = 2$ or $i = 3$; see Text F for the proof. In the fourth type of event, either node v_ℓ or $v_{\ell+i-1}$, which is of type A, is selected as parent; this event occurs with probability $2r/(ri + N - i)$. Then, the hyperedge that contains the parent and two nodes of type B is used for reproduction, which occurs with probability 1/3. In this case, state i increases by two. For example, if v_ℓ is selected as parent and hyperedge $\{\ell-2, \ell-1, \ell\}$ is used for reproduction, then the state moves from i to $i+2$. Therefore, we obtain

$$p_{i,i+2} = \frac{2r}{3(ri + N - i)}, \tag{S25}$$

which is Eq. (33) in the main text. If any other event occurs, then i remains unchanged. Therefore, we obtain

$$p_{i,i} = 1 - p_{i,i-2} - p_{i,i-1} - p_{i,i+1} - p_{i,i+2}, \tag{S26}$$

which is Eq. (34) in the main text.

Text D. Proof of Eq. (27) for $N = 4$

If $N = 4$, then $v_{\ell-2}$ and $v_{\ell+2}$ are identical. In this case, there are two sequences of events through which the state moves from $i = N - 1$ to $i = N$. In the first sequence, $v_{\ell-2}$, which is of type A, is selected as parent with probability $r/(3r + 1)$. Then, the hyperedge that contains the parent and v_ℓ, i.e., $\{\ell - 2, \ell - 1, \ell\}$ or $\{\ell, \ell + 1, \ell - 2\}$, is used for reproduction, which occurs with probability 2/3. In the second sequence, either $v_{\ell-1}$ or $v_{\ell+1}$, which is of type A, is selected as parent with probability $2r/(3r + 1)$. Then, one of the two hyperedges that contain the parent and v_ℓ is used for reproduction, which occurs with probability 2/3. By summing up these probabilities, we obtain Eq. (27).

Text E. Proof of Eq. (31) for $i = N - 3$ and $i = N - 2$

If $i = N - 3$, then $v_{\ell-2}$ and $v_{\ell+i+1}$ are identical. In this case, there are two sequences of events through which the state decreases from i to $i - 1$. In the first sequence, either $v_{\ell-1}$ or $v_{\ell-3}$ is selected as parent, which occurs with probability $2/(rN - 3r + 3)$. Then, the hyperedge containing two nodes of type B (i.e., hyperedge $\{\ell-2, \ell-1, \ell\}$ if $v_{\ell-1}$ is the parent and hyperedge $\{\ell-4, \ell-3, \ell-2\}$ if $v_{\ell-3}$ is the parent) is used for reproduction, which occurs with probability 1/3. In the second sequence, $v_{\ell-2}$ is selected as parent with probability $1/(rN - 3r + 3)$. Then, hyperedge $\{\ell-2, \ell-1, \ell\}$ or $\{\ell-4, \ell-3, \ell-2\}$ is used for reproduction, which occurs with probability 2/3. By summing up these probabilities, we obtain Eq. (31).

If $i = N - 2$, either of the two nodes of type B, i.e., $v_{\ell-1}$ or $v_{\ell-2}$, must be selected as parent for the state to move from i to $i - 1$. This event occurs with probability $2/(rN - 2r + 2)$. Then, either hyperedge $\{\ell-2, \ell-1, \ell\}$ or $\{\ell-3, \ell-2, \ell-1\}$ must be used for reproduction, which occurs with probability 2/3. The product of these two probabilities yields Eq. (31).

Text F. Proof of Eq. (32) for $i = 2$ and $i = 3$

If $i = 2$, for the state to move from i to $i + 1$, either v_ℓ or $v_{\ell+1}$, which is of type A, must be selected as parent. This event occurs with probability $2r/(2r + N - 2)$. Then, a hyperedge containing both v_ℓ and $v_{\ell+1}$ must be selected, which occurs with probability 2/3. The product of these two probabilities yields Eq. (32).

If $i = 3$, then $v_{\ell+1}$ and $v_{\ell+i-2}$ are identical. In this case, there are two sequences of events with which the state increases from i to $i + 1$. In the first sequence, either v_ℓ or $v_{\ell+2}$ is selected as parent with probability $2r/(3r + N - 3)$. Then, the hyperedge composed of the parent, $v_{\ell+1}$, which is of type A, and a node of type B (i.e., $v_{\ell-1}$ if the parent is v_ℓ, and $v_{\ell+3}$ if the parent is $v_{\ell+2}$) is used for reproduction with probability 1/3. In the second sequence, $v_{\ell+1}$ is selected as parent with probability $r/(3r + N - 3)$. Then, hyperedge $\{\ell - 1,\ \ell,\ \ell + 1\}$ or $\{\ell + 1,\ \ell + 2,\ \ell + 3\}$ is used for reproduction with probability 2/3. By summing up these probabilities, we obtain Eq. (32).

Text G. Derivation of the fixation probability for the star 3-uniform hypergraph under model 1

In this section, we derive the fixation probability for the star 3-uniform hypergraph under model 1.

Assume that there are currently i nodes of type A and that the state is $(i_1, i_2) = (1, i - 1)$ with $i \in \{1, \ldots, N - 1\}$. There are five types of events that can occur next.

In the first type of event, a leaf node of type B is selected as parent, which occurs with probability $(N - i)/(ri + N - i)$. Then, a hyperedge that contains the parent, the hub node, and a different leaf node of type B is used for reproduction, which occurs with probability $(N - i - 1)/(N - 2)$. The state after this entire event is $(i_1, i_2) = (0, i - 1)$ with $i \in \{1, \ldots, N - 2\}$. Therefore, we obtain

$$p_{(1,i-1)\to(0,i-1)} = \frac{N - i}{ri + N - i} \frac{N - i - 1}{N - 2}. \tag{S27}$$

In the second type of event, a leaf node of type B is selected as parent with probability $(N - i)/(ri + N - i)$. Then, a hyperedge that contains the parent, the hub node, and a leaf node of type A is used for reproduction,

which occurs with probability $(i-1)/(N-2)$. The state after this event is $(0, i-2)$ with $i \in \{2, \ldots, N-1\}$. Therefore, we obtain

$$p_{(1,i-1)\to(0,i-2)} = \frac{N-i}{ri+N-i}\frac{i-1}{N-2}. \tag{S28}$$

In the third type of event, the hub node, which is of type A, is selected as parent, which occurs with probability $r/(ri+N-i)$. Then, a hyperedge that contains the parent, a leaf node of type A, and a leaf node of type B is used for reproduction, which occurs with probability $(i-1)(N-i)/\binom{N-1}{2}$. Alternatively, a leaf node of type A is selected as parent, which occurs with probability $r(i-1)/(ri+N-i)$. Then, a hyperedge that contains the parent, the hub node, and a leaf node of type B is used for reproduction, which occurs with probability $(N-i)/(N-2)$. In both cases, the state after the event is $(1, i)$ with $i \in \{2, \ldots, N-1\}$. Therefore, we obtain

$$p_{(1,i-1)\to(1,i)} = \frac{r}{ri+N-i}\frac{(i-1)(N-i)}{\binom{N-1}{2}} + \frac{r(i-1)}{ri+N-i}\frac{N-i}{N-2}. \tag{S29}$$

In the fourth type of event, the hub node is selected as parent with probability $r/(ri+N-i)$. Then, a hyperedge that contains the parent and two leaf nodes of type B is used for reproduction, which occurs with probability $\binom{N-i}{2}/\binom{N-1}{2}$. The state after this event is $(1, i+1)$ with $i \in \{1, \ldots, N-2\}$. Therefore, we obtain

$$p_{(1,i-1)\to(1,i+1)} = \frac{r}{ri+N-i}\frac{\binom{N-i}{2}}{\binom{N-1}{2}}. \tag{S30}$$

If any other event occurs, then the state remains unchanged. Therefore, we obtain

$$p_{(1,i-1)\to(1,i-1)} = 1 - p_{(1,i-1)\to(0,i-1)} - p_{(1,i-1)\to(0,i-2)} - p_{(1,i-1)\to(1,i)} - p_{(1,i-1)\to(1,i+1)}. \tag{S31}$$

We recall $\tilde{x}_{(i_1,i_2)}$ is the probability that type A fixates starting with state (i_1, i_2). We obtain

$$\begin{aligned}\tilde{x}_{(1,i-1)} =& p_{(1,i-1)\to(0,i-1)}\tilde{x}_{(0,i-1)} + p_{(1,i-1)\to(0,i-2)}\tilde{x}_{(0,i-2)} + p_{(1,i-1)\to(1,i)}\tilde{x}_{(1,i)} \\ &+ p_{(1,i-1)\to(1,i+1)}\tilde{x}_{(1,i+1)} + p_{(1,i-1)\to(1,i-1)}\tilde{x}_{(1,i-1)}.\end{aligned} \tag{S32}$$

Assume that the current state is $(i_1, i_2) = (0, i)$ with $i \in \{1, \ldots, N-1\}$. There are five types of events that can occur next.

In the first type of event, a leaf node of type A is selected as parent with probability $ri/(ri+N-i)$. Then, a hyperedge that contains the parent, the hub node, and a different leaf node of type A is used for reproduction with probability $(i-1)/(N-2)$. The state after this entire event is $(i_1, i_2) = (1, i)$ with $i \in \{2, \ldots, N-1\}$. Therefore, we obtain

$$p_{(0,i)\to(1,i)} = \frac{ri}{ri+N-i}\frac{i-1}{N-2}. \tag{S33}$$

In the second type of event, a leaf node of type A is selected as parent with probability $ri/(ri+N-i)$. Then, a hyperedge that contains the parent, the hub node, and a leaf node of type B is used for reproduction, which occurs with probability $(N-i-1)/(N-2)$. The state after this event is $(1, i+1)$ with $i \in \{1, \ldots, N-2\}$. Therefore, we obtain

$$p_{(0,i)\to(1,i+1)} = \frac{ri}{ri+N-i}\frac{N-i-1}{N-2}. \tag{S34}$$

In the third type of event, the hub node, which is of type B, is selected as parent, with probability $1/(ri+N-i)$. Then, a hyperedge that contains the parent, a leaf node of type A, and a leaf node of type B, is used for reproduction, which occurs with probability $i(N-i-1)/\binom{N-1}{2}$. Alternatively, a leaf node of type B is selected as parent with probability $(N-i-1)/(ri+N-i)$. Then, a hyperedge that contains the parent, the hub node, and a leaf node of type A is used for reproduction, which occurs with probability $i/(N-2)$. In both cases, the state after the event is $(0, i-1)$ with $i \in \{1, \ldots, N-2\}$. Therefore, we obtain

$$p_{(0,i)\to(0,i-1)} = \frac{1}{ri+N-i}\frac{i(N-i-1)}{\binom{N-1}{2}} + \frac{N-i-1}{ri+N-i}\frac{i}{N-2}. \tag{S35}$$

In the fourth type of event, the hub node is selected as parent with probability $1/(ri + N - i)$. Then, the hyperedge that contains the parent and two leaf nodes of type A is used for reproduction, which occurs with probability $\binom{i}{2}/\binom{N-1}{2}$. The state after this event is $(0, i-2)$ with $i \in \{2, \dots, N-1\}$. Therefore, we obtain

$$p_{(0,i)\to(0,i-2)} = \frac{1}{ri + N - i} \frac{\binom{i}{2}}{\binom{N-1}{2}}. \tag{S36}$$

If any other event occurs, then the state remains unchanged. Therefore, we obtain

$$p_{(0,i)\to(0,i)} = 1 - p_{(0,i)\to(1,i)} - p_{(0,i)\to(1,i+1)} - p_{(0,i)\to(0,i-1)} - p_{(0,i)\to(0,i-2)}. \tag{S37}$$

Using these transition probabilities, we obtain

$$\begin{aligned} \tilde{x}_{(0,i)} =& p_{(0,i)\to(1,i)}\tilde{x}_{(1,i)} + p_{(0,i)\to(1,i+1)}\tilde{x}_{(1,i+1)} + p_{(0,i)\to(0,i-1)}\tilde{x}_{(0,i-1)} \\ &+ p_{(0,i)\to(0,i-2)}\tilde{x}_{(0,i-2)} + p_{(0,i)\to(0,i)}\tilde{x}_{(0,i)}. \end{aligned} \tag{S38}$$

We rewrite Eqs. (S32) and (S38) as

$$\tilde{\boldsymbol{x}} = P\tilde{\boldsymbol{x}}, \tag{S39}$$

which is Eq. (37) in the main text, where

$$P = \left(\begin{array}{c|c} C & D \\ \hline E & F \end{array}\right), \tag{S40}$$

which is Eq. (38) in the main text,

$$C = \begin{pmatrix} 1 & 0 & 0 & \cdots & 0 & 0 & 0 \\ p_{(0,1)\to(0,0)} & p_{(0,1)\to(0,1)} & 0 & \cdots & 0 & 0 & 0 \\ p_{(0,2)\to(0,0)} & p_{(0,2)\to(0,1)} & p_{(0,2)\to(0,2)} & \cdots & 0 & 0 & 0 \\ \vdots & \vdots & \vdots & \cdots & \vdots & \vdots & \\ 0 & 0 & 0 & \cdots & p_{(0,N-1)\to(0,N-3)} & p_{(0,N-1)\to(0,N-2)} & p_{(0,N-1)\to(0,N-1)} \end{pmatrix}, \tag{S41}$$

$$D = \begin{pmatrix} 0 & 0 & 0 & 0 & \cdots & 0 & 0 & 0 \\ 0 & p_{(0,1)\to(1,1)} & p_{(0,1)\to(1,2)} & 0 & \cdots & 0 & 0 & 0 \\ 0 & 0 & p_{(0,2)\to(1,2)} & p_{(0,2)\to(1,3)} & \cdots & 0 & 0 & 0 \\ \vdots & \vdots & \vdots & \vdots & \cdots & \vdots & \vdots & \\ 0 & 0 & 0 & 0 & \cdots & 0 & p_{(0,N-2)\to(1,N-2)} & p_{(0,N-2)\to(1,N-1)} \\ 0 & 0 & 0 & 0 & \cdots & 0 & 0 & p_{(0,N-1)\to(1,N-1)} \end{pmatrix}, \tag{S42}$$

$$E = \begin{pmatrix} p_{(1,0)\to(0,0)} & 0 & 0 & \cdots & 0 & 0 & 0 \\ p_{(1,1)\to(0,0)} & p_{(1,1)\to(0,1)} & 0 & \cdots & 0 & 0 & 0 \\ 0 & p_{(1,2)\to(0,1)} & p_{(1,2)\to(0,2)} & \cdots & 0 & 0 & 0 \\ \vdots & \vdots & \vdots & \cdots & \vdots & \vdots & \vdots \\ 0 & 0 & 0 & \cdots & p_{(1,N-2)\to(0,N-3)} & p_{(1,N-2)\to(0,N-2)} & 0 \\ 0 & 0 & 0 & \cdots & 0 & p_{(1,N-1)\to(0,N-2)} & p_{(1,N-1)\to(0,N-1)} \end{pmatrix}, \tag{S43}$$

and

$$F = \begin{pmatrix} p_{(1,0)\to(1,0)} & p_{(1,0)\to(1,1)} & p_{(1,0)\to(1,2)} & 0 & \cdots & 0 & 0 & 0 \\ 0 & p_{(1,1)\to(1,1)} & p_{(1,1)\to(1,2)} & p_{(1,1)\to(1,3)} & \cdots & 0 & 0 & 0 \\ \vdots & \vdots & \vdots & \vdots & \cdots & \vdots & \vdots & \vdots \\ 0 & 0 & 0 & 0 & \cdots & 0 & p_{(1,N-2)\to(1,N-2)} & p_{(1,N-2)\to(1,N-1)} \\ 0 & 0 & 0 & 0 & \cdots & 0 & 0 & 1 \end{pmatrix}. \tag{S44}$$

Text H. Fixation probability for the star 3-uniform hypergraph with $N = 1500$

In Fig A, we compare the fixation probability for the star 3-uniform hypergraph having $N = 1500$ nodes with that for the Moran process under model 1. The figure indicates that the star 3-uniform hypergraph with $N = 1500$ is a suppressor of selection.

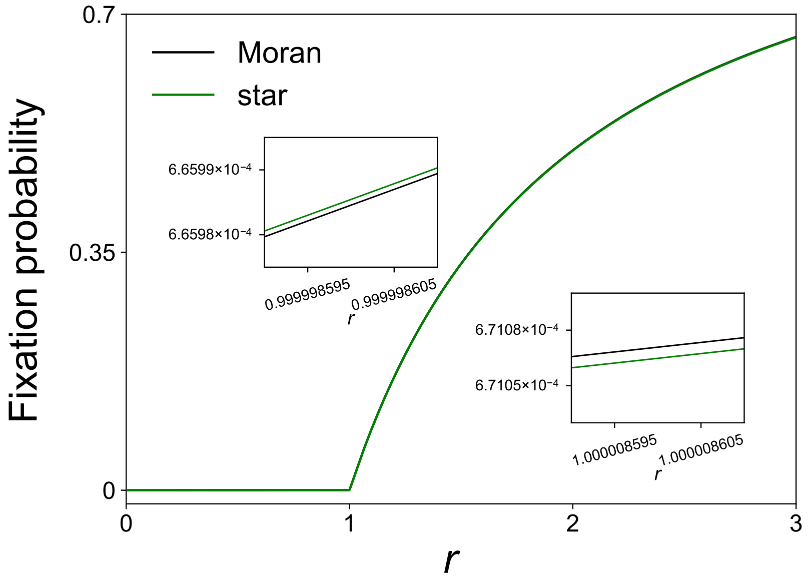

Fig A. Fixation probability for the star 3-uniform hypergraph with $N = 1500$. We compare it with the fixation probability for the Moran process. The inset on the left magnifies the result for r values smaller than and close to $r = 1$. The inset on the right magnifies the result for r values greater than and close to $r = 1$. In the main plot, the result for the Moran process, shown by the black line, is not identical but close to that for the star 3-uniform hypergraph, shown by the green line, such that the former is hidden behind the latter.

Text I. Fixation probability for the birth-death process on the weighted one-mode projection of the star 3-uniform hypergraph

In this section, we consider the weighted one-mode projection of the star 3-uniform hypergraph and examine the fixation probability on the obtained weighted network.

We denote the weighted one-mode projection of the star 3-uniform hypergraph by G. Note that G is a weighted complete graph; the edge between the hub node and any leaf node has weight $N - 2$, and the edge between any pair of leaf nodes has weight 1. In the birth-death process on G, in each time step, we select one node as parent with the probability proportional to its fitness. Then, the parent selects one of its neighbors with the probability proportional to the edge weight and converts the neighbor into the parent's type. As is the case for the star 3-uniform hypergraph, the symmetry in G allows us to specify the state of the birth-death process by tuple (i_1, i_2), where $i_1 \in \{0, 1\}$ specifies whether the hub is of type A (i.e., $i_1 = 1$) or B (i.e., $i_1 = 0$), and $i_2 \in \{0, 1, \ldots, N - 1\}$ is the number of leaf nodes of type A. The total number of

nodes of type A is equal to $i = i_1 + i_2$. The fixation of type A and B corresponds to $(i_1, i_2) = (1, N-1)$ and $(0, 0)$, respectively.

Assume that the current state is $(i_1, i_2) = (1, i-1)$ with $i - 1 \in \{0, 1, \ldots, N-2\}$. There are four types of events that can occur in the next time step. In the first type of event, a leaf node of type B is selected as parent with probability $(N-i)/(ri+N-i)$. Then, the edge between the parent and the hub node is used for reproduction with probability $1/2$. The state after this entire event is $(i_1, i_2) = (0, i-1)$. Therefore, we obtain

$$p_{(1,i-1)\to(0,i-1)} = \frac{N-i}{ri+N-i} \cdot \frac{1}{2}. \tag{S45}$$

In the second type of event, a leaf node of type B is selected as parent with probability $(N-i)/(ri+N-i)$. Then, the edge between the parent and a leaf node of type A is used for reproduction with probability $(i-1)/2(N-2)$. The state after this event is $(1, i-2)$. Therefore, we obtain

$$p_{(1,i-1)\to(1,i-2)} = \frac{N-i}{ri+N-i} \cdot \frac{i-1}{2(N-2)}. \tag{S46}$$

In the third type of event, the hub node, which is of type A, is selected as parent with probability $r/(ri+N-i)$. Then, the edge between the parent and a leaf node of type B is used for reproduction with probability $(N-i)/(N-1)$. Alternatively, a leaf node of type A is selected as parent with probability $r(i-1)/(ri+N-i)$. Then, the edge between the parent and a leaf node of type B is used for reproduction with probability $(N-i)/2(N-2)$. In both cases, the state after the event is $(1, i)$. Therefore, we obtain

$$p_{(1,i-1)\to(1,i)} = \frac{r}{ri+N-i} \cdot \frac{N-i}{N-1} + \frac{r(i-1)}{ri+N-i} \cdot \frac{N-i}{2(N-2)}. \tag{S47}$$

If any other event occurs, then the state remains unchanged. Therefore, we obtain

$$p_{(1,i-1)\to(1,i-1)} = 1 - p_{(1,i-1)\to(0,i-1)} - p_{(1,i-1)\to(1,i-2)} - p_{(1,i-1)\to(1,i)}. \tag{S48}$$

We remind that $\tilde{x}_{(i_1,i_2)}$ represents the probability that A fixates starting with state (i_1, i_2). We obtain

$$\tilde{x}_{(1,i-1)} = p_{(1,i-1)\to(0,i-1)}\tilde{x}_{(0,i-1)} + p_{(1,i-1)\to(1,i-2)}\tilde{x}_{(1,i-2)} + p_{(1,i-1)\to(1,i)}\tilde{x}_{(1,i)} + p_{(1,i-1)\to(1,i-1)}\tilde{x}_{(1,i-1)}. \tag{S49}$$

Now we assume that the current state is $(i_1, i_2) = (0, i)$ with $i \in \{1, 2, \ldots, N-1\}$. There are four types of events that can occur in the next time step. In the first type of event, a leaf node of type A is selected as parent with probability $ri/(ri+N-i)$. Then, the edge between the parent and the hub node is used for reproduction with probability $1/2$. The state after this event is $(i_1, i_2) = (1, i)$. Therefore, we obtain

$$p_{(0,i)\to(1,i)} = \frac{ri}{ri+N-i} \cdot \frac{1}{2}. \tag{S50}$$

In the second type of event, a leaf node of type A is selected as parent with probability $ri/(ri+N-i)$. Then, the edge between the parent and a leaf node of type B is used for reproduction with probability $(N-i-1)/2(N-2)$. The state after this event is $(0, i+1)$. Therefore, we obtain

$$p_{(0,i)\to(0,i+1)} = \frac{ri}{ri+N-i} \cdot \frac{N-i-1}{2(N-2)}. \tag{S51}$$

In the third type of event, the hub node, which is of type B, is selected as parent with probability $1/(ri+N-i)$. Then, the edge between the parent and a leaf node of type A is used for reproduction with probability $i/(N-1)$. Alternatively, a leaf node of type B is selected as parent with probability $(N-i-1)/(ri+N-i)$. Then, the edge between the parent and a leaf node of type A is used for reproduction with probability $i/2(N-2)$. In both cases, the state after the event is $(0, i-1)$. Therefore, we obtain

$$p_{(0,i)\to(0,i-1)} = \frac{1}{ri+N-i} \cdot \frac{i}{N-1} + \frac{N-i-1}{ri+N-i} \cdot \frac{i}{2(N-2)}. \tag{S52}$$

If any other event occurs, then the state remains unchanged. Therefore, we obtain

$$p_{(0,i)\to(0,i)} = 1 - p_{(0,i)\to(1,i)} - p_{(0,i)\to(0,i+1)} - p_{(0,i)\to(0,i-1)}. \tag{S53}$$

Using these transition probabilities, we obtain

$$\tilde{x}_{(0,i)} = p_{(0,i)\to(1,i)}\tilde{x}_{(1,i)} + p_{(0,i)\to(0,i+1)}\tilde{x}_{(0,i+1)} + p_{(0,i)\to(0,i-1)}\tilde{x}_{(0,i-1)} + p_{(0,i)\to(0,i)}\tilde{x}_{(0,i)}. \tag{S54}$$

Equation (37) also holds true for the one-mode projection. We use the scipy DGESV algorithm to numerically solve Eq. (37) to obtain $\tilde{x}_{(0,i)}$ and $\tilde{x}_{(1,i-1)}$. Then, we obtain x_i using Eq. (39).

We computed x_1 by numerically solving Eq. (37) for $N = 4, 5, 20$, and 200. Fig B compares the obtained x_1 values with those for the Moran process and the star 3-uniform hypergraph under model 1. We find that the one-mode projection of the star 3-uniform hypergraph is a weak amplifier of selection. Note that, for $N = 4$, 5, and 200, the results for the one-mode projection almost overlap those for the Moran process such that the orange lines are hidden behind the black lines in Fig BA, BB, and BD.

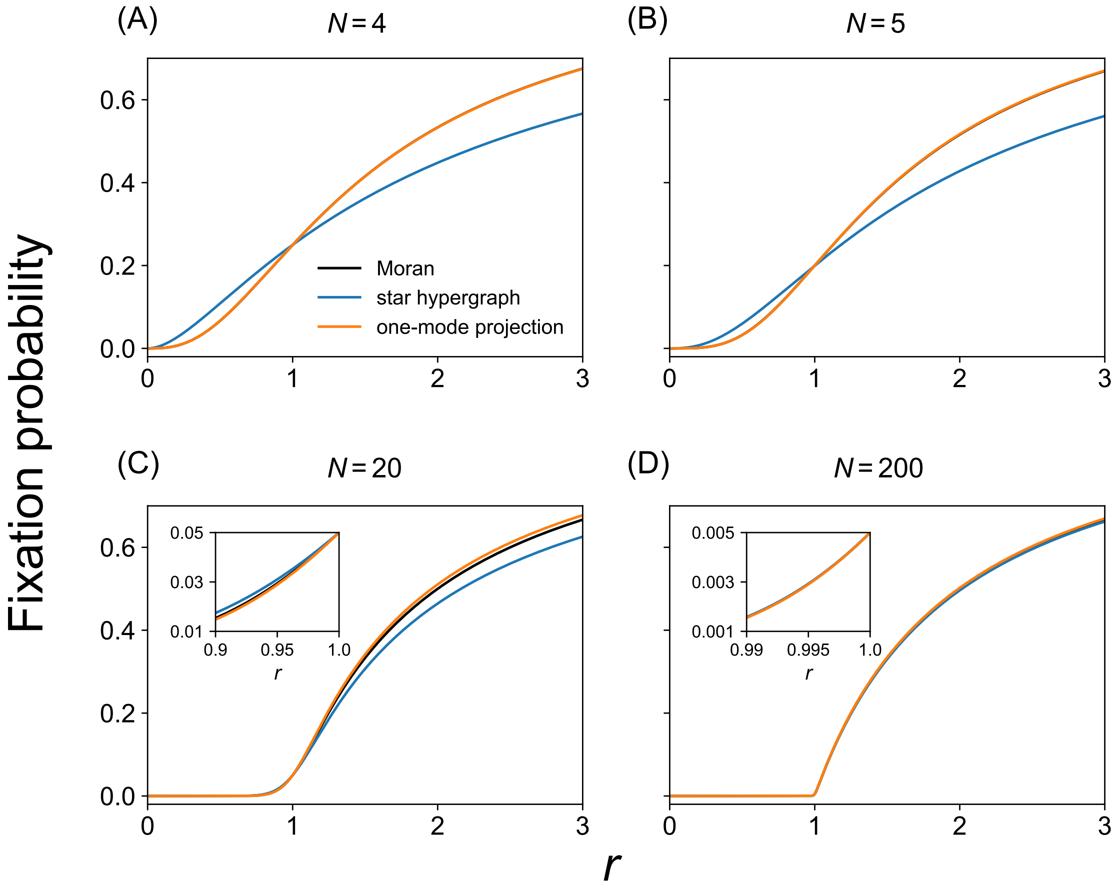

Fig B. Fixation probability for the weighted one-mode projection of star 3-uniform hypergraphs. We compare it with the fixation probability for the Moran process and star 3-uniform hypergraphs. (A) $N = 4$. (B) $N = 5$. (C) $N = 20$. (D) $N = 200$. The insets in (C) and (D) magnify the results for r values smaller than and close to $r = 1$. In the inset in (D), the results for the star 3-uniform hypergraph (shown by the blue line) and the Moran process (shown by the black line) are close to that for the one-mode projection (shown by the orange line) such that the blue and the black lines are almost hidden behind the orange line. In (A), (B), and the main panel of (D), the results for the Moran process (shown by the black lines) are not identical but close to those for the one-mode projection (shown by the orange lines) such that the former are hidden behind the latter.

Text J. Derivation of the fixation probability for the cyclic 3-uniform hypergraph under model 2

In this section, we derive the fixation probability for the cyclic 3-uniform hypergraph under model 2. We assume that there are initially just two mutants that are uniformly distributed.

The fixation of type A can occur only when the two nodes that are initially of type A share at least one hyperedge. Once such a hyperedge is selected for reproduction, all the nodes of type A are consecutive along the cycle without being interrupted by nodes of type B. Note that the two nodes that initially have type A may be next to each other on the cycle already in the initial condition. Therefore, to calculate the fixation probability on the cyclic 3-uniform hypergraph, it suffices to track the number of consecutive nodes having type A, which we denote by i. For $N \geq 5$, the initial condition is either of the following three types.

First type of initial condition

In the first type of initial condition, the two nodes of type A do not share any hyperedge (see Fig CA for a schematic), which occurs with probability $N(N-5)/\left[2\binom{N}{2}\right]$. The probability that type A fixates under this initial condition, denoted by x_2', is given by

$$x_2' = 0. \tag{S55}$$

Second type of initial condition

In the second type of initial condition, the two nodes of type A share two hyperedges, i.e., these two nodes are next to each other on the cycle (see Fig CB). This initial condition occurs with probability $N/\binom{N}{2}$. Let x_2'' be the fixation probability for type A under this initial condition. In general, let x_i'' with $i \in \{1, 2, \ldots, N-1\}$ be the fixation probability for type A when there are i consecutive nodes of type A and all the other nodes are of type B. We calculate x_2'' by tracking the number of consecutive nodes with type A, i.e., i, as follows.

Move of the state from i to $i-1$: If $i \in \{2, \ldots, N-2\}$, there are three types of events that can occur next. Without loss of generality, we assume that the ℓth to the $(\ell+i-1)$th nodes are of type A and that all the other nodes are of type B (see Fig 3C). In the first type of event, the state moves from i to $i-1$. If $i \leq N-4$, either $v_{\ell-2}$, $v_{\ell-1}$, $v_{\ell+i}$, and $v_{\ell+i+1}$, which is of type B, is selected as parent with probability $4/(ri+N-i)$. Then, the hyperedge that contains the parent node, a node of type B, and a node of type A is used for reproduction with probability 1/3. In this case, i decreases by one. For example, if $v_{\ell-2}$ is selected as parent and hyperedge $\{\ell-2, \ell-1, \ell\}$ is used for reproduction, then the state moves from i to $i-1$. Therefore, we obtain

$$p_{i,i-1} = \frac{4}{ri+N-i} \cdot \frac{1}{3}. \tag{S56}$$

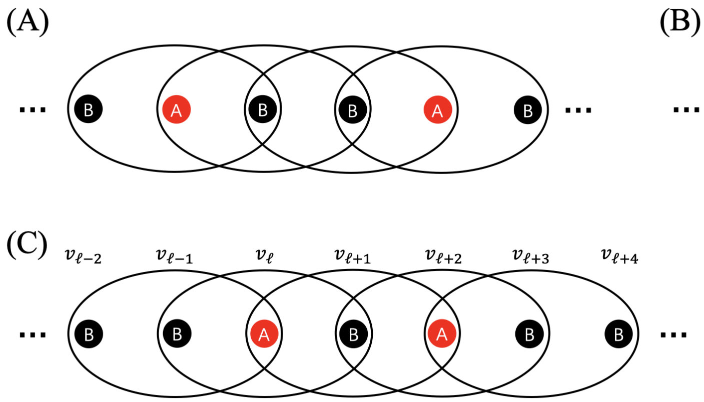

Fig C. Initial position of the two nodes of type A on the cyclic 3-uniform hypergraph. (A) The two nodes of type A do not share any hyperedge. (B) The two nodes of type A share two hyperedges. (C) The two nodes of type A share one hyperedge.

If $i = N-3$, there are two sequences of events through which the state decreases from i to $i-1$. In the first sequence, either $v_{\ell-1}$ or $v_{\ell-3}$ is selected as parent, which occurs with probability $2/(rN-3r+3)$. Then, the hyperedge containing two nodes of type B (i.e., hyperedge $\{\ell-2, \ell-1, \ell\}$ if $v_{\ell-1}$ is the parent and hyperedge $\{\ell-4, \ell-3, \ell-2\}$ if $v_{\ell-3}$ is the parent) is used for reproduction, which occurs with probability 1/3. In the second sequence, $v_{\ell-2}$ is selected as parent with probability $1/(rN-3r+3)$. Then, hyperedge $\{\ell-2, \ell-1, \ell\}$ or $\{\ell-4, \ell-3, \ell-2\}$ is used for reproduction, which occurs with probability 2/3. By summing up these probabilities, we obtain Eq. (S56). If $i = N-2$, either of the two nodes of type B, i.e., $v_{\ell-1}$ or $v_{\ell-2}$, must be selected as parent for the state to move from i to $i-1$. This event occurs with probability $2/(rN-2r+2)$. Then, either hyperedge $\{\ell-2, \ell-1, \ell\}$ or $\{\ell-3, \ell-2, \ell-1\}$ must be used for reproduction, which occurs with probability 2/3. The product of these two probabilities coincides with Eq. (S56). Therefore, Eq. (S56) holds true for any $i \in \{2, \ldots, N-2\}$.

Move of the state from i to $i+1$: In the second type of event, the state moves from i to $i+1$. If $i \geq 4$, either v_ℓ, $v_{\ell+1}$, $v_{\ell+i-2}$, or $v_{\ell+i-1}$, which is of type A, has to be selected as parent with probability $4r/(ri+N-i)$. Then, the hyperedge that contains the parent node, a node of type A, and a node of type B has to be used for reproduction, which occurs with probability 1/3. For example, if v_ℓ is selected as parent and hyperedge $\{\ell-1, \ell, \ell+1\}$ is used for reproduction, then the state moves from i to $i+1$. Therefore, we obtain

$$p_{i,i+1} = \frac{4r}{ri+N-i} \cdot \frac{1}{3}. \tag{S57}$$

If $i = 3$, there are two sequences of events through which the state increases from i to $i+1$. In the first sequence, either v_ℓ or $v_{\ell+2}$ is selected as parent, which occurs with probability $2r/(3r+N-3)$. Then, the hyperedge containing two nodes of type A (i.e., hyperedge $\{\ell-1,\ \ell,\ \ell+1\}$ if v_ℓ is the parent and hyperedge $\{\ell+1,\ \ell+2,\ \ell+3\}$ if $v_{\ell+2}$ is the parent) is used for reproduction, which occurs with probability 1/3. In the second sequence, $v_{\ell+1}$ is selected as parent with probability $r/(3r+N-3)$. Then, hyperedge $\{\ell-1,\ \ell,\ \ell+1\}$ or $\{\ell+1,\ \ell+2,\ \ell+3\}$ is used for reproduction, which occurs with probability 2/3. If we sum these probabilities, we obtain Eq. (S57). If $i = 2$, either of the two nodes of type A, i.e., v_ℓ or $v_{\ell+1}$, must be selected as parent for the state to move from i to $i+1$. This event occurs with probability $2r/(2r+N-2)$. Then, either hyperedge $\{\ell-1,\ \ell,\ \ell+1\}$ or $\{\ell,\ \ell+1,\ \ell+2\}$ must be used for reproduction, which occurs with probability 2/3. The product of these two probabilities coincides with Eq. (S57). Therefore, Eq. (S57) holds true for any $i \in \{2, \ldots, N-2\}$.

No move of the state from i: Because i remains unchanged if any other event occurs, we obtain

$$p_{i,i} = 1 - p_{i,i-1} - p_{i,i+1}. \tag{S58}$$

Derivation of x''_2: Therefore, the fixation probability of type A starting from i consecutive nodes of type A, i.e., x''_i, satisfies

$$x_0 = x_1 = 0, \tag{S59}$$

$$x''_i = p_{i,i-1}x''_{i-1} + p_{i,i}x''_i + p_{i,i+1}x''_{i+1}, \quad i \in \{2, \ldots, N-2\}, \tag{S60}$$

$$x_{N-1} = x_N = 1. \tag{S61}$$

Note that $x''_1 = x_1$ and $x''_{N-1} = x_{N-1}$. Similar to the analysis of the fixation probability for the complete 3-uniform hypergraph, we set

$$\overline{y}_i \equiv x''_i - x''_{i-1}, \quad i \in \{2, \ldots, N-1\}. \tag{S62}$$

Note that $\sum_{i=2}^{N-1} \overline{y}_i = x''_{N-1} - x''_1 = 1$. Let

$$\overline{\gamma}_i = p_{i,i-1}/p_{i,i+1}. \tag{S63}$$

By combining Eqs. (S58), (S60), (S62), and (S63), we obtain

$$\overline{y}_{i+1} = \overline{y}_i \overline{\gamma}_i, \tag{S64}$$

which leads to

$$\overline{y}_i = \overline{y}_2 \prod_{k=2}^{i-1} \overline{\gamma}_k = x_2'' \prod_{k=2}^{i-1} \overline{\gamma}_k. \tag{S65}$$

Using Eq. (S65), we obtain

$$\begin{aligned} 1 = \sum_{i=2}^{N-1} \overline{y}_i &= x_2'' \left[1 + \overline{\gamma}_2 + \overline{\gamma}_2\overline{\gamma}_3 + \cdots + \prod_{k=2}^{N-2} \overline{\gamma}_k \right] \\ &= x_2'' \left[1 + r^{-1} + r^{-2} + \cdots + r^{-(N-3)} \right] \\ &= x_2'' \frac{1 - r^{-(N-2)}}{1 - r^{-1}}. \end{aligned} \tag{S66}$$

Therefore, we obtain

$$x_2'' = \frac{1 - r^{-1}}{1 - r^{-(N-2)}}. \tag{S67}$$

Third type of initial condition

In the third type of initial condition, the two nodes of type A share one hyperedge, implying that there is a node of type B between the two nodes of type A. Without loss of generality, we assume that the ℓth and the $(\ell+2)$th nodes are of type A and that all the other nodes are of type B (see Fig CC). This initial condition, which we denote by 2^*, occurs with probability $N/\binom{N}{2}$. Now we calculate the fixation probability for type A starting from state 2^*, which we denote by x_2'''. To ease the discussion, in the remainder of this section, we denote by i the state in which consecutive i nodes on the cycle are of type A and the other $N-i$ nodes are of type B.

Move of the state from 2^* to $i = 1$: If $N \geq 7$, the state moves from 2^* to $i = 1$ in one time step if either of the following two types of events occurs. In the first type of event, either node $v_{\ell-2}$ or $v_{\ell+4}$, which is of type B, is selected as parent. This event occurs with probability $2/(2r + N - 2)$. Then, the hyperedge that contains the parent, a node of type B, and a node of type A, is used for reproduction, which occurs with probability $1/3$. For example, if $v_{\ell-2}$ is the parent and hyperedge $\{\ell-2, \ell-1, \ell\}$ is used for reproduction, then the state moves from 2^* to $i = 1$. In the second type of event, one of the nodes $v_{\ell-1}$, $v_{\ell+1}$, and $v_{\ell+3}$, which is of type B, is selected as parent, which occurs with probability $3/(2r + N - 2)$. Then, one of the two hyperedges that contains the parent, a node of type B, and a node of type A, is used for reproduction, which occurs with probability $2/3$. For example, if $v_{\ell-1}$ is selected as parent and hyperedge $\{\ell-2, \ell-1, \ell\}$ or $\{\ell-1, \ell, \ell+1\}$ is used for reproduction, then the state moves from 2^* to $i = 1$. By summing up these probabilities, we obtain the probability that the state moves from 2^* to $i = 1$ as

$$p_{2^*,1} = \frac{1}{2r + N - 2} \cdot \frac{8}{3}. \tag{S68}$$

If $N = 6$, the state moves from 2^* to $i = 1$ in one time step if the following event occurs. Either $v_{\ell-2}$, $v_{\ell-1}$, $v_{\ell+1}$, and $v_{\ell+3}$, which is of type B, is selected as parent with probability $4/(2r+4)$. Then, one of the two hyperedges that contains the parent, a node of type B, and a node of type A, is used for reproduction, which occurs with probability $2/3$. For example, if $v_{\ell-2}$ is selected as parent and hyperedge $\{\ell-4, \ell-3, \ell-2\}$ or $\{\ell-2, \ell-1, \ell\}$ is used for reproduction, then the state moves from 2^* to $i = 1$. The product of these two probabilities coincides with Eq. (S68).

If $N = 5$, the state moves from 2^* to $i = 1$ in one time step if either of the following two types of events occurs. In the first type of event, either node $v_{\ell-1}$ or $v_{\ell+3}$, which is of type B, is selected as parent with probability $2/(2r + 3)$. Then, the hyperedge that contains the parent, a node of type B, and a node of type A, is used for reproduction, which occurs with probability 1. For example, if $v_{\ell-1}$ is the parent and any hyperedge that contains $v_{\ell-1}$ is used for reproduction, then the state moves from 2^* to $i = 1$. In the second type of event, the node $v_{\ell+1}$, which is of type B, is selected as parent with probability $1/(2r + 3)$. Then, one of the two hyperedges $\{\ell-1, \ell, \ell+1\}$ or $\{\ell+1, \ell+2, \ell+3\}$ is used for reproduction, which occurs with probability $2/3$. By summing up these probabilities, we obtain Eq. (S68). Therefore, Eq. (S68) holds true for any $N \geq 5$.

Move of the state from 2^* to $i = 3$: The state moves from 2^* to $i = 3$ if either v_ℓ or $v_{\ell+2}$, which is of type A, is selected as parent with probability $2r/(2r + N - 2)$, and then, the hyperedge that contains v_ℓ, $v_{\ell+1}$, and $v_{\ell+2}$ is used for reproduction with probability 1/3. Therefore, we obtain

$$p_{2^*,3} = \frac{2r}{2r + N - 2} \cdot \frac{1}{3}. \tag{S69}$$

No move of the state from 2^*: If any other event occurs at state 2^*, the state remains unchanged. Therefore, we obtain

$$p_{2^*,2^*} = 1 - p_{2^*,1} - p_{2^*,3} = \frac{4r + 3N - 14}{3(2r + N - 2)}, \tag{S70}$$

$$p_{2^*,j} = 0 \text{ if } j \neq 1, 2^*, 3. \tag{S71}$$

Derivation of x_2''': If the state moves from 2^* to either $i = 1$ or $i = 3$, all the nodes of type A are consecutively numbered without being interrupted by nodes of type B afterwards. Therefore, we obtain

$$x_2''' = p_{2^*,1}x_1 + p_{2^*,2^*}x_2''' + p_{2^*,3}x_3''. \tag{S72}$$

By substituting Eqs. (S59) and (S67) in Eq. (S60) for $i = 2$, we obtain

$$x_3'' = \frac{1 - r^{-2}}{1 - r^{-(N-2)}}. \tag{S73}$$

By substituting Eqs. (S59) and (S73) in Eq. (S72), we obtain

$$x_2''' = \frac{r - r^{-1}}{(r + 4)\left[1 - r^{-(N-2)}\right]}. \tag{S74}$$

Weighted sum to obtain x_2

By combining Eqs. (S55), (S67), and (S74) with the respective probability, we obtain the fixation probability for type A when there are initially two uniformly randomly distributed mutants, given in Eq. (70), as follows:

$$\begin{aligned} x_2 &= \frac{N-5}{N-1}x_2' + \frac{2}{N-1}x_2'' + \frac{2}{N-1}x_2''' \\ &= \frac{2}{N-1}\left\{\frac{1 - r^{-1}}{1 - r^{-(N-2)}} + \frac{r - r^{-1}}{(r+4)\left[1 - r^{-(N-2)}\right]}\right\}, \end{aligned} \tag{S75}$$

where $N \geq 5$.

Derivation of x_2 for $N = 4$

For $N = 4$, the first type of initial condition occurs with probability 0.

The second type of initial condition occurs with the same probability as in the case of $N \geq 5$, i.e., with probability $4/\binom{4}{2} = 2/3$. In this case, the state moves from 2 to 1 if the following event occurs. Either $v_{\ell-1}$ or $v_{\ell+2}$, which is of type B, is selected as parent with probability $2/(2r + 2)$. Then, either hyperedge $\{\ell+2, \ell-1, \ell\}$ or $\{\ell+1, \ell+2, \ell-1\}$ must be used for reproduction, which occurs with probability 2/3. Note that the node indices $\ell - 1$ and ℓ are equivalent to $\ell + 3$ and $\ell + 4$ because we interpret the node index with modulo N. The product of these two probabilities coincides with Eq. (S56). Therefore, Eq. (S56) also holds true for $N = 4$. Similarly, Eq. (S57) holds true for $N = 4$. Therefore, using Eq. (S66), we obtain

$$x_2'' = \frac{r}{r + 1}. \tag{S76}$$

Under the third type of initial condition, which occurs with probability $2/\binom{4}{2} = 1/3$, the state moves from 2^* to 1 if the following event occurs. Either $v_{\ell-1}$ or $v_{\ell+1}$, which is of type B, is selected as parent

with probability $2/(2r+2)$. Then, either hyperedge $\{\ell-1,\ell,\ell+1\}$ or $\{\ell+1,\ell+2,\ell-1\}$ must be used for reproduction, which occurs with probability $2/3$. Therefore, we obtain

$$p_{2^*,1} = \frac{2}{3r+3}. \tag{S77}$$

The state moves from 2^* to 3 if the following event occurs. Either v_ℓ or $v_{\ell+2}$, which is of type A, is selected as parent with probability $2r/(2r+2)$. Then, either hyperedge $\{\ell,\ell+1,\ell+2\}$ or $\{\ell+2,\ell-1,\ell\}$ must be used for reproduction, which occurs with probability $2/3$. Therefore, we obtain

$$p_{2^*,3} = \frac{2r}{3r+3}. \tag{S78}$$

If any other event occurs at state 2^*, the state remains unchanged. Therefore, we obtain

$$p_{2^*,2^*} = 1 - p_{2^*,1} - p_{2^*,3} = \frac{r+1}{3r+3}, \tag{S79}$$

$$p_{2^*,j} = 0 \text{ if } j \neq 1, 2^*, 3. \tag{S80}$$

By substituting Eqs. (S59) and (S61) in Eq. (S72), we obtain

$$x_2^{'''} = \frac{r}{r+1}. \tag{S81}$$

Therefore, we obtain

$$x_2 = \frac{2}{3}x_2^{''} + \frac{1}{3}x_2^{'''} = \frac{r}{r+1} \tag{S82}$$

for $N = 4$.

Text K. Derivation of the fixation probability for the star 3-uniform hypergraph under model 2

We derive the fixation probability for the star 3-uniform hypergraph under model 2 in this section. We use same notations as those in section 3.1.3.

Assume that the current state is $(i_1, i_2) = (1, i-1)$ with $i-1 \in \{0, 1, \ldots, N-2\}$. There are three types of events that can occur in the next time step. In the first type of event, a leaf node of type B is selected as parent with probability $(N-i)/(ri+N-i)$. Then, a hyperedge that contains the parent, the hub node, and a different leaf node of type B, is used for reproduction with probability $(N-i-1)/(N-2)$. The state after this entire event is $(i_1, i_2) = (0, i-1)$. Therefore, we obtain

$$p_{(1,i-1)\to(0,i-1)} = \frac{N-i}{ri+N-i} \cdot \frac{N-i-1}{N-2}. \tag{S83}$$

In the second type of event, the hub node, which is of type A, is selected as parent with probability $r/(ri+N-i)$. Then, a hyperedge that contains the parent, a leaf node of type A, and a leaf node of type B, is used for reproduction with probability $(i-1)(N-i)/\binom{N-1}{2}$. Alternatively, a leaf node of type A is selected as parent with probability $r(i-1)/(ri+N-i)$. Then, a hyperedge that contains the parent, the hub node, and a leaf node of type B, is used for reproduction with probability $(N-i)/(N-2)$. In both cases, the state after the event is $(1, i)$. Therefore, we obtain

$$p_{(1,i-1)\to(1,i)} = \frac{r}{ri+N-i} \cdot \frac{(i-1)(N-i)}{\binom{N-1}{2}} + \frac{r(i-1)}{ri+N-i} \cdot \frac{N-i}{N-2}. \tag{S84}$$

If any other event occurs, then the state remains unchanged. Therefore, we obtain

$$p_{(1,i-1)\to(1,i-1)} = 1 - p_{(1,i-1)\to(0,i-1)} - p_{(1,i-1)\to(1,i)}. \tag{S85}$$

We remind that $\tilde{x}_{(i_1,i_2)}$ is the probability that type A fixates when the initial state is (i_1, i_2). We obtain

$$\tilde{x}_{(1,i-1)} = p_{(1,i-1)\to(0,i-1)}\tilde{x}_{(0,i-1)} + p_{(1,i-1)\to(1,i)}\tilde{x}_{(1,i)} + p_{(1,i-1)\to(1,i-1)}\tilde{x}_{(1,i-1)}. \tag{S86}$$

Now we assume that the current state is $(i_1, i_2) = (0, i)$ with $i \in \{1, \ldots, N-1\}$. Then, there are three types of events that can occur in the next time step. In the first type of event, a leaf node of type A is selected as parent with probability $ri/(ri + N - i)$. Then, a hyperedge that contains the parent, the hub node, and a different leaf node of type A, is used for reproduction with probability $(i-1)/(N-2)$. The state after this event is $(i_1, i_2) = (1, i)$. Therefore, we obtain

$$p_{(0,i)\to(1,i)} = \frac{ri}{ri + N - i} \cdot \frac{i-1}{N-2}. \tag{S87}$$

In the second type of event, the hub node, which is of type B, is selected as parent with probability $1/(ri + N - i)$. Then, a hyperedge that contains the parent, a leaf node of type A, and a leaf node of type B, is used for reproduction with probability $i(N-i-1)/\binom{N-1}{2}$. Alternatively, a leaf node of type B is selected as parent with probability $(N-i-1)/(ri+N-i)$. Then, a hyperedge that contains the parent, the hub node, and a leaf node of type A, is used for reproduction with probability $i/(N-2)$. In both cases, the state after the event is $(0, i-1)$. Therefore, we obtain

$$p_{(0,i)\to(0,i-1)} = \frac{1}{ri + N - i} \cdot \frac{i(N-i-1)}{\binom{N-1}{2}} + \frac{N-i-1}{ri + N - i} \cdot \frac{i}{N-2}. \tag{S88}$$

If any other event occurs, then the state remains unchanged. Therefore, we obtain

$$p_{(0,i)\to(0,i)} = 1 - p_{(0,i)\to(1,i)} - p_{(0,i)\to(0,i-1)}. \tag{S89}$$

Using these transition probabilities, we obtain

$$\tilde{x}_{(0,i)} = p_{(0,i)\to(1,i)}\tilde{x}_{(1,i)} + p_{(0,i)\to(0,i-1)}\tilde{x}_{(0,i-1)} + p_{(0,i)\to(0,i)}\tilde{x}_{(0,i)}. \tag{S90}$$

Equations (S86) and (S90) lead to

$$\tilde{x}_{(1,i)} = \alpha_i \tilde{x}_{(1,i-1)} + (1 - \alpha_i)\tilde{x}_{(0,i-1)} \tag{S91}$$

and

$$\tilde{x}_{(0,i)} = \beta_i \tilde{x}_{(1,i)} + (1 - \beta_i)\tilde{x}_{(0,i-1)}, \tag{S92}$$

respectively, where

$$\alpha_i = 1 + \frac{(N-i-1)(N-1)}{r(i-1)(N+1)}, \tag{S93}$$

$$\beta_i = \frac{r(i-1)(N-1)}{r(i-1)(N-1) + (N-i-1)(N+1)}. \tag{S94}$$

We rewrite Eqs. (S91) and (S92) as

$$\boldsymbol{\varrho_i} = A_i \boldsymbol{\varrho_{i-1}}, \tag{S95}$$

where $\boldsymbol{\varrho_i} = (\tilde{x}_{(1,i)}, \tilde{x}_{(0,i)})^\top$, and A_i is the 2×2 matrix given by

$$A_i = \begin{pmatrix} \alpha_i & 1 - \alpha_i \\ \alpha_i\beta_i & 1 - \alpha_i\beta_i \end{pmatrix}. \tag{S96}$$

Equation (S95) yields

$$\boldsymbol{\varrho_i} = A_i A_{i-1} \cdots A_2 \boldsymbol{\varrho_1}. \tag{S97}$$

Therefore,

$$\begin{pmatrix} 1 \\ 1 \end{pmatrix} = \boldsymbol{\varrho_{N-1}} = A_{N-1}A_{N-2}\cdots A_2\boldsymbol{\varrho_1}. \tag{S98}$$

Equation (39) also holds true for model 2. Therefore, we obtain $\tilde{x}_{(1,1)}$ from Eq. (S98), $\tilde{x}_{(0,2)}$ from Eq. (S95), and finally x_2 from Eq. (39).

By analytically solving Eq. (S98) for $N = 4$ and $N = 5$, we obtain Eqs. (55) and (56), respectively.
